# Platelet-concentrated and platelet poor-plasma promote different pattern on immunohistochemical expression of TGF-β1, however they impairs the osteoneogensis in calvarial defects treated with autograft due suppression of osteocalcin

**DOI:** 10.1590/ACB360604

**Published:** 2021-07-19

**Authors:** Marco Antonio de Oliveira-Filho, Marcelo Souza, Fernando Issamu Tabushi, Luís Eduardo Almeida, Fernanda Pirajá Figueiredo, Elora Sampaio Lourenço, Allan Fernando Giovanini, Osvaldo Malafaia, Jurandir Marcondes Ribas

**Affiliations:** 1 PhD. Medical Research Institute - Hospital Universitário Evangélico Mackenzie – Curitiba (PR), Brazil.; 2 MsC. Medical Research Institute - Hospital Universitário Evangélico Mackenzie – Curitiba (PR), Brazil.; 3 PhD. Medical Research Institute - Hospital Universitário Evangélico Mackenzie – Curitiba (PR), Brazil.; 4 PhD. Assistant Professor. Marquette University - United States.; 5 Graduate student. Hospital Universitário Evangélico Mackenzie – Curitiba (PR), Brazil.; 6 Graduate student. Hospital Universitário Evangélico Mackenzie – Curitiba (PR), Brazil.; 7 PhD. Medical Research Institute - Hospital Universitário Evangélico Mackenzie – Curitiba (PR), Brazil.; 8 PhD. Medical Research Institute - Hospital Universitário Evangélico Mackenzie – Curitiba (PR), Brazil.; 9 PhD. Medical Research Institute - Hospital Universitário Evangélico Mackenzie – Curitiba (PR), Brazil.

**Keywords:** Bone Regeneration, Osteocalcin, Transforming Growth Factor beta1, Rabbits

## Abstract

**Purpose:**

Herein we evaluated the effects of platelet concentrate (PC) and platelet-poor plasma (PPP) on bone repair using noncritical defects in the calvaria of rabbits and compared them to the presence of TGF-β1 and osteocalcin on reparative sites.

**Methods:**

Five noncritical defects of 8.7 mm in diameter were created on the calvaria of 15 animals. Each defect was treated differently, using autograft (ABG), ABG associated with PC (ABG + PC), ABG with PPP (ABG + PPP), isolated PPP, and blood clot (control). The animals were submitted to euthanasia on the second, fourth and sixth week post-surgery.

**Results:**

The defects that received ABG+PC or PPP demonstrated lower bone formation when compared to specimens that received ABG in the same period. These results coincided to significant higher immunopositivity for TGF-β1 for specimens that received PC, and lower presence of cytokine in the group PPP. However, either higher or lower presence of TGF-β1 were also correlated to lower presence of osteocalcin. Likewise, these results were similar to findings in specimens treated only with PPP when compared to control.

**Conclusions:**

PC and PPP were not effective when applied in association with ABG. Similarly, isolated use of PPP was not beneficial in optimizing the bone repair.

## Introduction

It is an agreement in the literature that the use of autogenous particulate bone graft (ABG) has been considered the gold standard among graft materials for bone repair in craniofacial bone^1^. Because there are limited amounts of autograft available, researchers have suggested that the combination of autograft and nonimmunogenic biomaterials that may contribute to osteoconduction could be a likely alternative to produce adequate and faster bone repair^1^,^2^.

The platelet-rich plasma (PRP) constitutes the first generation of platelet concentrate (PC). This platelet concentrate is a blood product enriched with platelets and white cells in small amount of plasma obtained after immediate plasma centrifugation^3^. The premise about the use of PC on wound healing tissue is associated to the presence of platelets, that when activated release several amounts of growth factors that include PDGF, TGF-b, VEGF, IGF-1, and EGF^4^-^6^. Hypothetically, these growth factors, especially the TGF-b, may contribute to stem cells’ mesenchymal chemotaxis, synthesis, and secretion of collagen on extracellular matrix, as well as to proliferation and cell differentiation into osteoblast, increasing the expression of osteocalcin, favoring bone tissue regeneration and osteoconduction7,8. However, previous studies were not able to identify osteogenic action of PC when it was used alone to repair bone defects^7^,^9^.

The disagreement of a consistent result about the effect of PC on the osteogenesis has been discussed regarding the variations in the PRP production^5^. Herein, we produced and used a PC whose amount of platelets or leukocytes was not accessed. Thus, the centrifuged blood product used in this study may be only mentioned as PC, because it differs from Marx *et al*.’s^3^ protocol, that measured platelets at the same time that they removed leukocytes, or from Arnoczky *et al*.’s^10^ protocol, that also accessed the number of platelets. Their protocols included white blood cells (leukocytes) in their final product. However, it should be highlighted that the PC protocol used here corroborates with several protocols previously published in literature and constitutes the majority of PC used in clinical and surgical practices^5^,^9^.

On the other hand, the platelet-poor plasma (PPP) corresponds to a level of plasma containing few platelets. Differently from PC, PPP contains an intense amount of serum proteins (e.g., fibrin, fibronectin, and vitronectin) that are important for cell adhesion^11^. Actually, studies that have attempted to evaluate the effects of PPP in bone tissue are scarce and their results are uncertain. However, using an animal experimental study, Yilmaz *et al*.^12^ indicated PPP as a likely osteoinductive biomaterial, since they demonstrated the presence of PPP stimulates osteoblastic proliferation, increasing DNA synthesis and resulting in osteoblast proliferation.

Since neither PC nor PPP possess unanimous or robust results on craniofacial bone repair, herein we analyzed the osteoconduction effect stimulated by PC and PPP associated to autograft and PPP alone, in artificial bone defects created in rabbits calvaria. Yet, we compared the histological aspects of bone repair at two, four, and six weeks on immunohistochemical presence of TGF-ß1 and osteocalcin (OC) in order to understand the bone repair induced by PC and PPP.

## Methods

### Animal model

This study was performed at the Institute of Medical Research of the Hospital Universitário Evangélico Mackenzie, after approval of its Ethics Committee.

Fifteen New Zealand female rabbits aged between 350 and 370 days old, weighing 2,850–4,400 g (3,244 g ± 388 g), from the vivarium of the Institute of Medical Research, (IPEM) were used.

### Platelet concentrate and platelet-poor plasma preparation

**Figure 1:**
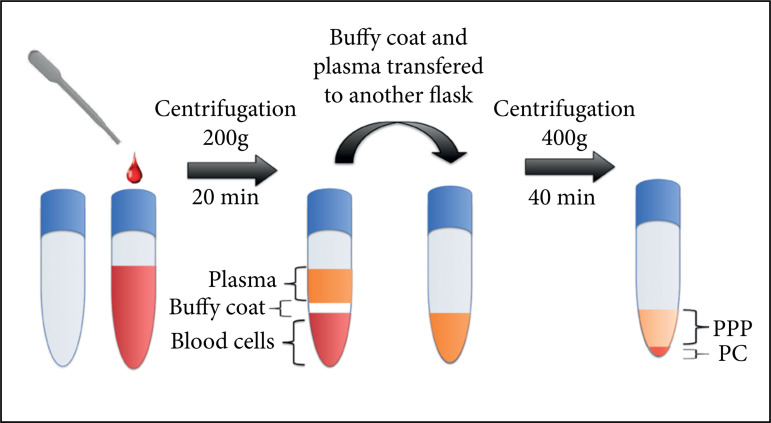
Schematic model of platelet-poor plasma and platelet concentrate preparation.

For the venous blood collection, the most favorable ear vein of each animal was punctioned using a scalp 21. Afterwards, a 10-mL syringe with 10% sodium citrate was connected to the scalp. Approximately 10 mL of blood from each rabbit was collected and transferred to a 160 × 100-mm tube. For the preparation of PC, a double-centrifugation technique was performed, as outlined in Fig. 1. At first, tubes containing blood material were centrifuged at 200 g for 20 min, allowing the formation of two distinct fractions: plasma at the superior part of the tube (slightly yellow colored), and the blood cells at the bottom (red colored). All of the plasma fraction plus the upper part (1 mL) of the blood cells fraction were transferred to another tube and submitted to a second cycle of 400 g for10 min. After this last cycle, two distinct fractions could be identified. The upper fraction was removed to the point in which its reminiscent plus the bottom fraction completed a total of 1 mL. After homogenization,1 mL of final product from the initial 10 mL of blood was obtained and used as PC. The uppermost portion removed was used as PPP.

### Sedation and surgical procedure

The experimental surgical process was performed according to previous study published by Oliveira-Filho *et al*.^9^. Thus, for the surgical approach, the animals were positioned inside a campanula individually, and sedation was promoted with oxygen and isoflurane (Cristália, Itapira, SP, Brazil) followed by an intramuscular injection on the posterior part of the thigh with 2.3-g xylazine (0.52 mg/kg) and 1.16-g ketamine (1.04 mg/kg) (Vetbrands, Paulínia, SP, Brazil). Herein, the anesthesia protocol was sustained with isoflurane vaporization (Cristália, Itapira, SP, Brazil) using a facial mask. After the anesthesia induction, shaving and antisepsis prepared with 1% polyvinylpyrrolidone iodine (PVPI) solution of active iodine of the areas of calvaria were carried out.

A midline dermoperiosteal incision of 5 cm was performed, raising a periosteal skin flap to expose the parietal bones surface with total removal of the periosteum using a curette for bone. Five noncritical defects (considered <10 mm in diameter in rabbits) measuring 8.7 mm in diameter ´ 2 mm in deep were created with a trephine drill (Biomedical Research Instruments, Silver Spring, MD, United States) under profuse saline solution irrigation.

Bone fragments that were removed from each calvarial defect were particulated and used as bone autografts. The fragmented bone obtained from the calvarial defects was created through an instrument for bone particulation developed by Neodent. A digital camera (Sony Cyber-shot DSC-w800, Tokyo, Japan) captured the images of the particles, whose average of particle size was obtained using ImageJ software (National Institutes of Health, Bethesda, MD, United States). An image size of 1 mm was used to standardize all measurements. Herein, the average particle size obtained was 882.52 ± 36.38 mm^2^.

For PC and PPP coagulation, a mixture of 10% calcium chloride solution and 5,000 units of bovine thrombin was added to the previously prepared PC and PPP(1 min for gel formation). One defect was grafted with autogenous particulate bone graft (ABG), [anterior face, right side], another with ABG associated with highly PC (ABG + PC) [anterior face, left side], ABG withPPP (ABG + PPP) [posterior face, right side], isolated PPP[posterior face, left side], and one defect had no grafting (control) [medial posterior face]. Tissue flaps of the wound were then closed in a running fashion.

For postoperative analgesia, the animals received morphine sulphate (3 mg/kg) (União Química, São Paulo, SP, Brazil) intramuscularly at the end of the surgery. Yet, the analgesia was maintained in postoperative period with 25 pills of paracetamol diluted into 500 mLof water placed into water drink for five days.

Histological performance

The animals were euthanatized with an overdose of the anesthetic solution after two weeks (five animals), four weeks (five animals), and six weeks (five animals). Block specimens were obtained using an inverted cone bur. The original surgical defect area and the surrounding tissues were removed in blocks. The pieces were fixed in 10% formalin for three days, and the surgical piece posteriorly was decalcified in 20% formic acid solution (Sigma-Aldrich, Darmstadt, Germany). Posteriorly, each piece was hemi-sectioned, perpendicularly to the sagittal suture. Longitudinal serial slices, measuring 5 mm of thickness, starting from the center of the original surgical defect, were obtained. The slices were stained with hematoxylin and eosin according the follow specification:

1. Deparaffinize sections, two changes of xylene, 15 minutes each;
2. Hydrate in two changes of absolute alcohol, 5 minutes each;
3. 95% alcohol for 2 minutes following for 70% alcohol for 2 minutes;
4. Wash briefly in distilled water;
5. Stain in Harris hematoxylin solution for 5 minutes;
6. Wash in running tap water for 5 minutes;
7. Differentiate in 1% acid alcohol for 10 seconds;
8. Wash running tap water for 1 minute;
9. Wash in running tap water for 5 minutes;
10. Rinse in 95% alcohol, 10 dips;
11. Counterstain in eosin solution for 20 seconds;
12. Dehydrate using two changes on 75% alcohol, two changes of 95% alcohol and two changes of absolute alcohol, 5 minutes each;
13. Clear in two changes of xylene, 5 minutes each;
14. Mount with xylene based on Permount mounting medium (Sigma-Aldrich, Darmstadt, Germany).

### Immunohistochemical procedure

Two-µm thicknesses of each sample were deparaffinized in xylene and hydrated for 10 minutes in absolute alcohol, following 10 min each in 95% alcohol and 70% alcohol. The antigen retrieval process was performed using 1% pepsin solution (pH 1.8) (Sigma-Aldrich, Darmstadt, Germany) for 1 hour at 37°C. They were allowed to cool to room temperature for 20 min and washed in distilled water for 10 min. Endogenous peroxidase activity was blocked in 0.1% hydrogen peroxide (Thermo Fisher Scientific, Waltham, MA, United States) for 15 min. The specimens were washed in running tap water for 5 minutes, and posteriorly submerged in phosphate-buffered saline (Sigma-Aldrich, Darmstadt, Germany) for 5 min.

Then, the samples were incubated overnight with the primary antibody anti-TGF-ß1 (200 mg/mL) (Santa Cruz Biotechnology, Santa Cruz, CA, United States), dilution factor of 1:200, and anti-OC (200 mg/mL) (Santa Cruz Biotechnology, Santa Cruz, CA, United States), dilution factor of 1:150. To detect the primary antibodies, a labeled streptavidin biotin antibody-binding detection system was used (Universal HRP immunostaining kit) (Diagnostic BioSystems, Foster City, CA, United States), for 30 min, and submitted to immersion with diaminobenzidine chromogen (Universal HRP immunostaining kit) (Diagnostic BioSystems, Foster City, CA, United States) for 15 min. It produced a brownish precipitate at the antigen site. The specimens were counterstained with Harris’s hematoxylin. A negative control was performed for all samples omitting primary antibody. For each specimen, three slides were utilized for incubation with each antibody.

### Histomorphometric and immunohistochemical analysis

Image acquiring was done with the use of a light microscope (21/3, Quimis Aparelhos Científicos, Diadema, SP, Brazil) and an SDC-310 camera (Samsung, Seongnam, South Korea), according to a previously published methodology^9^. Three randomly selected microscopic fields within each grafted area from all groups and animals were analyzed.

Histomorphometric parameters were analyzed using the UTHSCSA Image Tool 2.00 (University of Texas Health Science Center, Houston, TX, United States). A total of ˜ 10 mm^2^ was analyzed in each field. Data were recorded in mm^2^ for each parameter. Simultaneously, the percentage of positive TGF-ß1 and OC were calculated by automation system, following the protocol established by Di Cataldo *et al.* 13. This automated counting allowed counting only the percentage of protein present in the whole defect, but it was not possible to distinguish their immunopositivity for membrane, cytoplasm or extracellular matrix.

### Statistical analysis

The bone matrix deposition was evaluated within the monitoring period. A Shapiro-Wilk analysis was used to determine the normality, followed by the Kruskal-Wallis nonparametric test, to verify significant differences among groups. A significance level of 0.05 was used for all analyses. Data were analyzed using the Statistical Package for the Social Sciences (SPSS Statistics) v.20 computer program (IBM Corporation, Armonk, NY, United States).

## Results

### Microscopic analysis

The histological features for each group are demonstrated in Fig. 2. The quantitative data for the histomorphometric results for bone matrix presence in each group are given in Table 1. A concise description of the histological frame found in each group is provided ahead.

**Table 1:** Histomorphometric analysis among the groups regarding bone matrix formation*,**.

Time period			PPP	PPP+Autograft	PRP+Autograft	Autograft	Control
2 Weeks			2.91 ± 0,75	5.61 ± 0,87	6.08 ± 1.03	6.61 ± 1.46	2.84 ± 0.29
	PPP	2.91 ± 0.75	----	< 0.001	< 0.001	< 0.001	0.916
	ABG+PPP	5.61 ± 0.87		----	= 0.461	= 0.123	< 0.001
	ABG+PC	6.08 ± 1.03			----	= 0.397	< 0.001
	ABG	6.61 ± 1,.46				----	< 0.001
	Control	2.84 ± 029					----
			5.01 + 0.93	6.08 ± 1.69	6.0 ± 1.4	8.24 ± 1.18	5.27 ± 1.02
4 Weeks	PPP	5.01 ± 0.93	----	= 0.207	= 0.242	< 0.001	= 0.743
	ABG+PPP	6.08 ±1.69		----	= 0.922	= 0.018	= 0.341
	ABG+PC	6.0 ± 1.4			----	= 0.015	= 0.390
	ABG	8.24 ± 1.18				----	= 0.002
	Control	5.27 ± 1.02					----
			7.52 ± 0.64	7.73 ± 1.41	7.53 ± 1.3	9.64 ± 1.43	7.86 ± 0.57
6 Weeks	PPP	7.52 ± 0.64	----	= 0.709	= 0.983	= 0.002	= 0.551
	ABG+PPP	7.73 ± 1.41		----	= 0,725	= 0.004	= 0.821
	ABG+PC	7.53 ± 1.3			----	= 0.002	= 0.565
	ABG	9.64 ± 1.43				----	= 0.006
	Control	7.86 ± 0.57					----

* Non-parametric Kruskal-Wallis test was employed;

** p < 0.05 indicates statistical significance; PPP: platelet-pour plasma; ABG: autogenous particulate bone graft;PC: platelet concentrate; PRP: platelet-rich plasma.

**Figrue 2:**
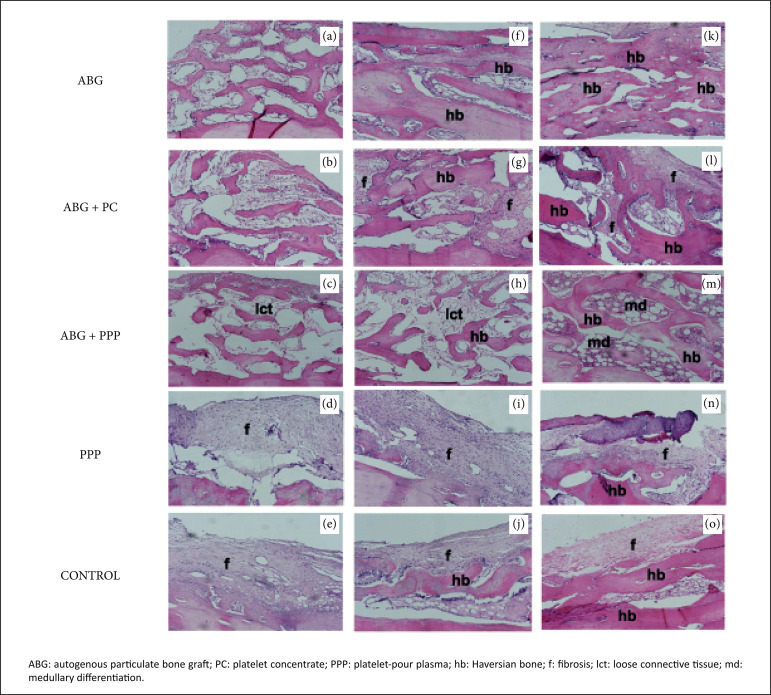
Micrographs **(a-e)** demonstrate the histological characteristics in the ABG, ABG + PC, ABG + PPP, PPP and control groups, respectively, two weeks after surgery. A, B and C reveal similar presence of autograph in the ABG, ABG + PC and ABG + PPP groups, while connective tissue was predominant in the PPP (D) and control (E) groups. Micrographs **(f-j)** demonstrate the same groups four weeks after surgery. The larger bone deposition on the defect treated with only ABG is evident, while the osteoneogenesis was lower for the groups that received PC and PPP. The patterns of the PPP and control groups at four weeks were similar, but in the control group there was a scarce presence of bone matrix in the host bone. These patterns were similar to the sixth week **(k-o)**. In this period, the intense presence of bone matrix in the ABG group may be seen. In the groups that received ABG + PC and ABG + PPP, the presence of osteogenesis was lower, while for the PPP and control groups the robust parts of the defects were composed of fibrotic tissue (hematoxylin and eosin stain, original magnification ×100).

#### Autogenous particulate bone graft

In the second week post-surgery, new bone formation exhibiting basophilic reversal lines suggesting osteoconductor activity was observed in area of defects. All new bone tissue formed was surrounded by connective tissue, composed of moderate cellularity fusiform. After four weeks, a substantial area of compact bone in the surgical bed was seen surrounding areas of scarce bone marrow area in the defects. Collagenous stromal tissue completed the histological frame in this period. After six weeks, predominantly mature and Haversian compact bone tissue was observed surrounding occasional area of medullary tissue.

#### Autogenous particulate bone graft + platelet concentrate

Two weeks after surgery, the inserted bone fragments were observed surrounded by a rich collagen matrix composed by cellularity fusiform. Areas compatible with areas of new bone formation exhibiting basophilic reversal lines suggesting osteoconductor activity were scarce. On the fourth and the sixth week, it was observed an evident connective tissue consistent with fibrosis surrounding the bone tissue that increased slowly, as soon as the period of time progressed.

#### Autogenous particulate bone graft + platelet-pour plasma

On the second week, it was observed that autograft fragments surrounded by loose connective tissue that was composed of thin and delicate collagen fibers poorly cellularized with fusiform cellular content. This pattern was also observed in the fourth week after surgery. However, the amount of bone matrix was larger when comparedto the anterior period. In the sixth week, it was observed a well-formed cancellous bone represented by Haversian bone matrix, as well as an intense well-formed medullar area that was composed of the exuberant presence of lymphoreticular cells and discreet fatty cells among the neoformed bone tissue.

#### Platelet-pour plasma

Microscopic analysis after two weeks revealed the presence of loose connective tissue with very few fusiform cells or new blood vessels. At four weeks, the histological pattern revealed a fibrotic tissue with scarce new bone formation derived from remaining host bone. At six weeks, the histological characteristics were similar. However, theamount of bone matrix deposition was larger when compared to four weeks.

#### Control

Histological analysis after two weeks revealed intense fibrous connective tissue compatible with reparative fibrosis, with very few fusiform cells or new blood vessels and reduced fatty cells. After four and six weeks, the histological patterns were similar and demonstrated an immaturebone tissue in the adjacent host bone area and remaining fibroustissue.

### Immunohistochemical analysis

The quantitative data for the histomorphometric results for bone matrix presence in each group are given in Tables 2 and 3, for TGF-ß1 and OC, respectively. A concise description of immunohistochemical data found for each group is provided ahead.

**Table 2:** Percentage of immunohistochemical presence of TGF-ß1 analysis among the groups*,**.

Time period			PPP	ABG+PPP	ABG + PC	ABG	Control
2 Weeks			12.65 ± 1.73	38.44 ± 2.61	76.05 ± 3.11	51.14 ± 2.38	17.22 ± 1.67
	PPP	12.65 ± 1.73	----	< 0.001	< 0.001	< 0.001	0.046
	ABG+PPP	38.44 ± 2.61		----	= 0.006	= 0.018	< 0.001
	ABG +PC	76.15 ± 3.11			----	= 0.048	< 0.001
	ABG	51.14 ± 2.38				----	< 0.001
	Control	17.22 ± 1.67					----
4 Weeks			3.84 ± 1.02	3.08 ± 1.69	76.08 ± 2.26	48.11 ± 1.74	45.27 ± 2.22
	PPP	3.84 ± 1.02	----	= 0.746	< 0.001	< 0.001	< 0.001
	ABG+PPP	3.08 ± 1.69		----	< 0.001	< 0.001	< 0.001
	ABG +PC	76.08 ± 2.26			----	= 0.015	= 0.028
	ABG	48.11 ± 1.74				----	= 0.704
	Control	45.27 ± 2.22					----
6 Weeks			7.42 ± 0.38	4.83 ± 1.66	69.82 ± 1.37	37.77 ± 2.17	48.21 ± 0.99
	PPP	7.42 ± 0.38	----	= 0.062	< 0.001	< 0.001	< 0.001
	ABG+PPP	4.83 ± 1.66		----	< 0.001	< 0.001	< 0.001
	ABG +PC	69.82 ± 1.37			----	= 0.024	= 0.003
	ABG	37.77 ± 2.17				----	= 0.066
	Control	48.21 ± 0.99					----

* Non-parametric Kruskal-Wallis test was employed;

** p< 0.05 indicates statistical significance; PPP: platelet-pour plasma; ABG: autogenous particulate bone graft; PC: platelet concentrate

**Table 3:** Percentage of immunohistochemical presence of osteocalcin analysis among the groups*,**.

Time period			PPP	ABG+PPP	ABG + PC	ABG	Control
2 Weeks			8.32 ± 0.42	26.19 ± 2.89	34.07 ± 2.51	76.89 ± 3.05	44.62 ± 1.18
	PPP	8.32 ± 0.42	----	< 0.001	< 0.001	< 0.001	0.046
	ABG+PPP	26.19 ± 2.89		----	= 0.624	< 0.001	= 0.002
	ABG +PC	34.07 ± 2.51			----	< 0.018	= 0.026
	ABG	76.89 ± 3.05				----	= 0.002
	Control	44.62 ± 1.18					----
4 Weeks			71.16 ± 2.33	17.11 ± 1.49	31.84 ± 2.83	71.16 ± 2.33	43.11 ± 1.94
	PPP	4.38 ± 0.87	----	= 0.046	< 0.001	< 0.001	< 0.001
	ABG+PPP	17.11 ± 1.49		----	< 0.018	< 0.001	< 0.001
	ABG +PC	31.84 ± 2.83			----	= 0.022	= 0.028
	ABG	71.16 ± 2.33				----	= 0.044
	Control	43.11 ± 1.94					----
6 Weeks			3.82 ± 0.19	14.28 ± 1.42	27.28 ± 1.66	69.08 ± 2.73	42.21 ± 0.99
	PPP	3.82 ± 0.19	----	= 0.006	< 0.001	< 0.001	< 0.001
	ABG+PPP	14.28 ± 1.42		----	= 0.018	< 0.001	< 0.001
	ABG +PC	27.28 ± 1.66			----	= 0.002	= 0.003
	ABG	69.08 ± 2.73				----	= 0.044
	Control	42.21 ± 0.99					----

Non-parametric Kruskal-Wallis test was employed;

** p< 0.05 indicates statistical significance; PPP: platelet-pour plasma; ABG: autogenous particulate bone graft; PC: platelet concentrate

#### TGF-ß1

**Figure 3:**
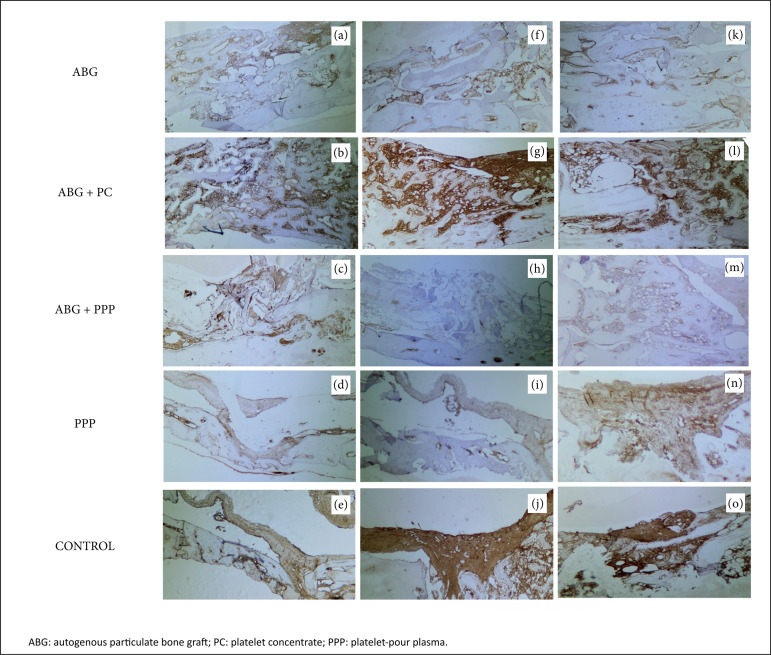
Micrographs **(a-e)** demonstrate the pattern of distribution of immunoexpression of TGF-ß1 (brownish color) in the ABG, ABG + PC, ABG + PPP, PPP and control groups, respectively, two weeks after surgery. Verify the intense presence of TGF-ß1 in ABG+PC group, while for the specimens in which PPP was inserted the immunopositivity for TGF-ß1 was scarce. Micrographs **(f-j)** demonstrate the same groups four weeks after surgery. The patterns of TGF-ß1 were suppressed in PPP groups, but the TGF-ß1 remains intense in specimens that received PC. These patterns were also similar to the sixth week **(k-o)**. However, it was verified that the cytokine increased in extravascular area in specimens that received PPP. Micrographs **(f-j)** demonstrate the same groups on the sixth week after surgery. Verify the similar pattern of distribution of osteocalcin when compared to earlier stage of bone repair (second week) (original magnification ×100).

On the second week, all groups analyzed demonstrated positivity to TGF-ß1, as may be seen in Fig. 3. In the groups filled with PC, the immunopositivity was intense, and the percentage of positivity of TGF-ß1 was considerably significant higher than in ABG and control groups. In contrast, the groups that received PPP in the areas positive for TGF-ß1 were scarce, and its value was lower when compared to ABG or control. On the fourth and sixth post-operative weeks, the pattern of TGF-ß1 was similar to groups ABG and ABG+PC. However, for specimens that received PPP, the immunopositivity for TGF-ß1 decreased, while in the group control the positivity for TGF-ß1 increased as soon as that fibrotic area was formed.

**Figure 4:**
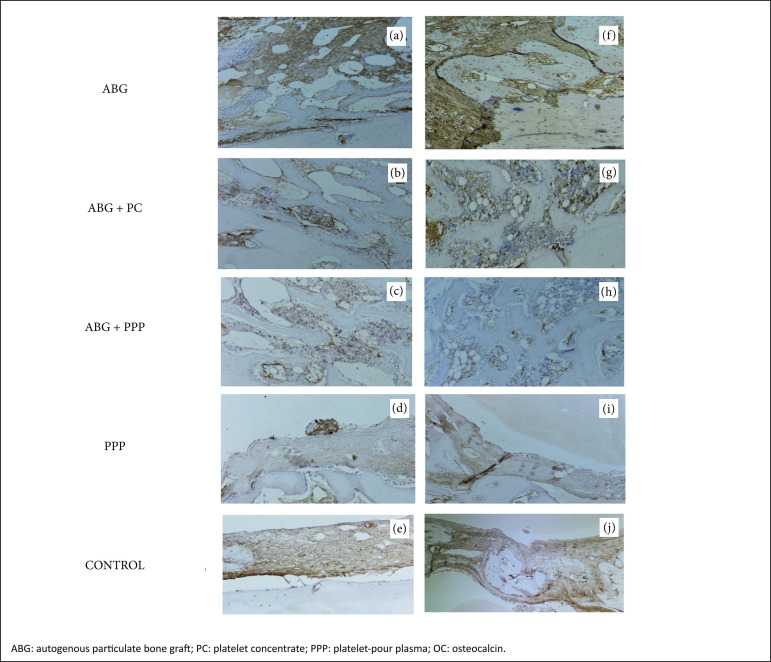
Micrographs **A, B, C, D** and **E** demonstrate the pattern of distribution of immunoexpression of OC (brownish color) in the ABG, ABG + PC, ABG + PPP, PPP and control groups, respectively, two weeks after surgery. Verify the intense presence of OC on ABG+PC group while in the specimens in which PC or PPP was inserted the immunopositivity for OC was fewer (*brownish color*). However, it was evident that specimens that were treated with PC revealed higher OC when compared to specimens that received PPP, associated or not to ABG (original magnification ×250).

## Discussion

Bone repair is a complex process that involves cell adhesion, proliferation, migration, differentiation, and synthesis and secretion of the peculiar extracellular matrix rich in collagen I, which possesses mineralization potential^14^.

It is necessary to emphasize that the approach of bone defects for study of bone healing may be heterogeneous^15^. However, the trauma in order to obtain surgical bed or on craniotomy approach may provide injuries, such as traumatic brain injury, as well as disruption of the blood-brain barrier integrity and the neurovascular unit, culminating in vascular leakage, edema, hemorrhage, hypoxia and neuronal death^16^. According to this premise, Özevren *et al*.^16^,^17^ demonstrated traumatic brain injury when cortical is injured or removed. The authors described a variety of pathological factors, such as oxidative stress, the inflammatory response, and apoptosis, as well as enlarged blood vessels, bleeding and swelling after traumatic injury in the brain and choroid plexus. On the other hand, Laçin *et al*.^18^ add that the presence of cortical or even its grafts provide a durable and rigid structure, condition that may give convenient model for evaluating bone regenerative effects of biomaterials. Herein we used the non-critical model created in rabbit calvaria, maintaining the cortical bone to assess the osteogenic effects of PC and PPP.

The use of PC in bone repair is based on the hypothesis that a significant number of platelets secrete an intense amount of active growth factors in response to granulation tissue at the injured site, especially collagen^7^. These growth factors seem to be responsible not only for proliferation, but also due to induction of differentiation of mesenchymal cells into osteoblasts^6^.

Although many studies have demonstrated a positive effect of PC on osteoneogenesis, others studies revealed deleterious results^19^. The lack of unanimous results about the effect of PC on bone regeneration is attributed to variations in the PC preparations that could alter the platelet quality and/or quantity, which would result in differences in the regenerative potential of PC^14^-^22^. The PC production method used in this study has proven to be able to concentrate large amounts of platelets (upward of 6× concentration), differing from many authors who proposed the hypothesis that intermediate concentrations of platelets could optimize the repair, with maximum stimulation effects generated with approximate concentrations of 2.5×, unlike what would occur with higher concentrations^9^,^23^.

Thus, distinct PC results could be correlated with this increase in platelets, whose action could stimulate a fibrous condition rather than osteogenesis, mimicking a pathological fibrosis effect simultaneously to the scarce bone matrix deposition, as found in the present study. This hypothesis is supported by Giovanini *et al*.^24^, who demonstrated a strong correlation between the presence of TGF-ß1 secreted by high-platelet concentration and a histological frame that mimicked a myelofibrosis. It should be highlighted that the intense production and positivity for TGF-ß1produced by platelets also decrease the expression of important proteins that acts on canonical and non-canonical pathways of osteogenesis, such as BMP2^21^, Wnt^1^ and osteoprotegerin levels^23^. Together, these hypotheses corroborate to results demonstrated herein and may explain the fewer amount of bone matrix development in areas in which ABP+PC was used, associated to exuberant positivity for TGF-ß1, while the presence of OC was scarce.

Differently from the idea stipulated for PC, previous studies have not identified several growth factors that possess proliferative capacity in PPP^25^. However, Cáceres *et al*.^26^ found that, even in minor TGF-ß1 concentrations (4.4 times lower when compared to PC), osteoblastic cells responded similarly to PPP and PC, showing that favorable responses could be achieved using low concentrations of TGF-ß1. Yet, Yilmaz *et al*.^12^ added that PPP may stimulate osteoblastic proliferation, increasing DNA synthesis and performing a mitogenic effect of osteoblasts, a condition that improves the osteoconductive effect.

Our results demonstrated that, in fact, the specimens that received PPP (associated or not to ABG) showed significant lower expression for TGF-ß1 when compared to other specimens. However, we also revealed that the osteogenesis was fewer when compared only to ABG, control or specimens that received PC. The result demonstrated herein differs of what was described by Cáceres *et al*.^26^ and Yilmaz *et al*.^12^. Herein we verified that the fewer bone formation coincided with concomitant scarce expression of both TGF-ß1 and OC in surgical area in which PPP was applied.

Yet, the results presented here revealed that, in the earliest stages of bone repair, PPP promoted larger quantities of thin and delicate extracellular matrix tissue added to autograft, while the majority of cells present in the regenerative sites were composed of fusiform cells. This result suggests that PPP induces cellular attachment, but the lack of growth factors suppresses the osteogenic differentiation in regenerative sites and favors a long period of usual medullary histophenotype, since it produces a stroma compatible with loose connective.

It is noteworthy that this cross-sectional study has some limitations. The analysis occurred only between two and six weeks in the postoperative period and no conclusions for immediate or latter effects of PC or PPP could be clarified. In addition, we used five defects produced in rabbit calvaria using distinct treatments. Under this context, we cannot evaluate the likely cross effect among the treatments neither cytokine action at a distance from the source that produced it.

Furthermore, there are many variables that generate doubts and need to be clarified for a safe use of autologous blood concentrates. For example, CP or PRPs have numerous growth factors, and the real interaction between them is unknown. There is also uncertainty as to whether these growth factors are competitive among them, or even if there is competition for a specific receptor to produce bone excitation or inhibition. Thus, as a future trend, further research is needed using a pool of growth factors which mimic the growth factors secreted by platelets, so they can be analyzed in different cell cultures to better understand how these factors interact (stimulating or inhibiting each other), and which factors act on cellular stimulation, expression of receptors or even as a transcription factor for osteogenic proteins.

## Conclusion

It may be concluded that neither PC nor PPP increased the bone formation when their applications were in association with ABG grafts. Similarly, isolated use of PPP was not beneficial in optimizing the bone repair.

## References

[1] Giovanini AF, Deliberador TM, Tannuri Nemeth JE, Crivellaro VR, Portela GS, Oliveira MA, de Araujo MR, Zielak JC, Gonzaga CC (2013). Leukocyte-platelet-rich plasma (L-PRP) impairs the
osteoconductive capacity of the autograft associated to changes in the
immunolocalization of TGF-?1 and its co-expression with Wnt10b and CD34
cells. J Craniomaxillofac Surg.

[2] Baldwin P, Li DJ, Auston DA, Mir HS, Yoon RS, Koval KJ. (2019). Autograft, allograft, and bone graft substitutes: clinical
evidence and indications for use in the setting of orthopaedic trauma
surgery. J Orthop Trauma.

[3] Marx RE, Carlson ER, Eichstaedt RM, Schimmele SR, Strauss JE, Georgeff KR (1998). Platelet-rich plasma: growth factor enhancement for bone
grafts. Oral Surg Oral Med Oral Pathol Oral Radiol Endod.

[4] Aghaloo TL, Moy PK, Freymiller EG. (2002). Investigation of platelet-rich plasma in rabbit cranial defects:
a pilot study. J Oral Maxillofac Surg.

[5] Roh YH, Kim W, Park K, Oh JH (2016). Cytokine-release kinetics of platelet-rich plasma according to
various activation protocols. Bone Joint Res.

[6] Messora MR, Nagata MJ, Dornelles RC, Bomfim SR, Furlaneto FA, de Melo LG, Deliberador TM, Bosco AF, Garcia VG, Fucini SE (2008). Bone healing in critical-size defects treated with platelet-rich
plasma activated by two different methods. A histologic and histometric
study in rat calvaria. J Periodontal Res.

[7] Giovanini AF, Deliberador TM, Gonzaga CC, de Oliveira MA, Göhringer I, Kuczera J, Zielak JC, de Andrade Urban C (2010). Platelet-rich plasma diminishes calvarial bone repair associated
with alterations in collagen matrix composition and elevated CD34+ cell
prevalence. Bone.

[8] Petrera M, De Croos JNA, Iu J, Hurtig M, Kandel RA, Theodoropoulos JS (2013). Supplementation with platelet-rich plasma improves the in vitro
formation of tissue-engineered cartilage with enhanced mechanical
properties. Arthroscopy.

[9] Oliveira MA, Nassif PA, Malafaia O, Ribas JM, Ribas CA, Camacho AC, Stieven E, Giovanini AF. (2010). Effects of a highly concentrated platelet-rich plasma on the bone
repair using non-critical defects in the calvaria of rabbits. Acta Cir Bras.

[10] Arnoczky SP, Delos D, Rodeo SA. (2011). What is platelet-rich plasma?. Operative techniques in sports Medicine.

[11] Cattaneo M, Lecchi A, Zighetti ML, Lussana F. (2007). Platelet aggregation studies: autologous platelet-poor plasma
inhibits platelet aggregation when added to platelet-rich plasma to
normalize platelet count. Haematologica.

[12] Yilmaz S, Kabadayi C, Ipci SD, Cakar G, Kuru B (2011). Treatment of intrabony periodontal defects with platelet-rich
plasma versus platelet-poor plasma combined with a bovine-derived xenograft:
a controlled clinical trial. J Periodontol.

[13] Di Cataldo, Ficarra E, Acquaviva A, Macii E. (2010). Automated segmentation of tissue images for computerized IHC
analysis. Comput Methods Programs Biomed.

[14] Marsell R, Einhorn TA. (2011). The biology of fracture healing. Injury.

[15] Kadiro?lu ET, Akbal?k ME, Karaöz E, Kanay BE, Da? A, Ketani MA, Ero?lu EG, Uysal E, Tuncer MC (2020). Calvarial bone defects in ovariectomised rats treated with
mesenchymal stem cells and demineralised freeze-dried bone
allografts. Folia Morphol (Warsz).

[16] Özevren H, Deveci E, Tuncer MC. (2020). The effect of rosmarinic acid on deformities occurring in brain
tissue by craniectomy method. Histopathological evaluation of IBA-1 and GFAP
expressions. Acta Cir Bras.

[17] Özevren H, Deveci E, Tuncer MC. (2018). Histopathological changes in the choroid plexus after traumatic
brain injury in the rats: a histologic and immunohistochemical
study. Folia Morphol (Warsz).

[18] Laçin N, İzol BS, Özkorkmaz EG, Deveci B, Tuncer MC. (2019). The effect of graft application and allopurinol treatment on
calvarial bone defect in rats. Acta Cir Bras.

[19] Intini G (2009). The use of platelet-rich plasma in bone reconstruction
therapy. Biomaterials.

[20] Nikolidakis D, Jansen JA. (2008). The biology of platelet-rich plasma and its application in oral
surgery: literature review. Tissue Eng Part B Rev.

[21] Giovanini AF, Grossi JR, Gonzaga CC, Zielak JC, Göhringer I, Vieira J, Kuczera J, de Oliveira MA, Deliberador TM (2014). Leukocyte-platelet-rich plasma (L-PRP) induces an abnormal
histophenotype in craniofacial bone repair associated with changes in the
immunopositivity of the hematopoietic clusters of differentiation,
osteoproteins, and TGF-?1. Clin Implant Dent Relat Res.

[22] Murray IR, LaPrade RF. (2016). Platelet rich plasma: renewed scientific understanding must guide
appropriate use. Bone Joint Res.

[23] Graziani F, Ivanovski S, Cei S, Ducci F, Tonetti M, Gabriele M. (2006). The in vitro effect of different PRP concentrations on
osteoblasts and fibroblasts. Clin Oral Implants Res.

[24] Giovanini AF, Gonzaga CC, Zielak JC, Deliberador TM, Kuczera J, Göringher I, de Oliveira MA, Baratto-Filho F, Urban CA. (2011). Platelet-rich plasma (PRP) impairs the craniofacial bone repair
associated with its elevated TGF-? levels and modulates the co-expression
between collagen III and ?-smooth muscle actin. J Orthop Res.

[25] Schnabel LV, Mohammed HO, Miller BJ, McDermott WG, Jacobson MS, Santangelo KS, Fortier LA. (2007). Platelet rich plasma (PRP) enhances anabolic gene expression
patterns in flexor digitorum superficialis tendons. J Orthop Res.

[26] Cáceres M, Martínez C, Martínez J, Smith PC (2012). Effects of platelet-rich and -poor plasma on the reparative
response of gingival fibroblasts. Clin Oral Implants Res.

[27] Rivera-Chacon DM, Alvarado-Velez M, Acevedo-Morantes CY, Singh SP, Gultepe E, Nagesha D, Sridhar S, Ramirez-Vick JE (2013). Fibronectin and vitronectin promote human fetal osteoblast cell
attachment and proliferation on nanoporous titanium surfaces. J Biomed Nanotechnol.

